# Functional assessments in the rodent stroke model

**DOI:** 10.1186/2040-7378-2-13

**Published:** 2010-07-19

**Authors:** Krystal L Schaar, Miranda M Brenneman, Sean I Savitz

**Affiliations:** 1 University of Texas Medical School at Houston, Department of Neurology, 6431 Fannin Houston, TX 77030, USA

## Abstract

Stroke is a common cause of permanent disability accompanied by devastating impairments for which there is a pressing need for effective treatment. Motor, sensory and cognitive deficits are common following stroke, yet treatment is limited. Along with histological measures, functional outcome in animal models has provided valuable insight to the biological basis and potential rehabilitation efforts of experimental stroke. Developing and using tests that have the ability to identify behavioral deficits is essential to expanding the development of translational therapies. The present aim of this paper is to review many of the current behavioral tests that assess functional outcome after stoke in rodent models. While there is no perfect test, there are many assessments that are sensitive to detecting the array of impairments, from global to modality specific, after stroke.

## Review

Stroke is a common and permanent cause of disability that is associated with sensory and motor deficits including lack of coordination and partial paralysis [1,2]. Additionally, higher cortical brain areas can be affected, and as a result, memory disturbances and other cognitive deficits often occur [2]. Due to the devastating impairments an individual can encounter after suffering a stroke, there is an immediate need for effective treatment. Rodents serve as an excellent model to explore the understanding and effects of human stroke among many other neural injuries. In addition to examining histopathological measures in rodents, it is important to assess functional outcome after stroke. The evaluation of neurological function allows for the assessment of the degree of damage over a period of time. The ultimate goal of stroke treatment is the restoration of behavioral function in patients. Identifying behavioral deficits and therapeutic treatments in animal models of stroke is essential for potential translational applications.

Although rodent models have provided valuable insight and understanding of the biological basis and functional outcome of stroke, the selection of individual tests is crucial for the success of translational research. It is imperative to choose tests that are sensitive to both the area of the brain damage and the interventions that are being applied. Due to the loss of limb function after stroke, many tests focus on motor and sensory tests. Since learning and memory impairments are also common after stroke, cognitive testing is also a crucial component in understanding the full scope of deficits. Therefore, it is important to have behavioral methods that are sensitive to detect the array of impairments occurring after stroke. Behavioral assessments also provide the opportunity to monitor pharmaceutical and cell-based treatments by observing functional improvements over time. With this in mind, it is ideal to have animal models that resemble the human model as closely as possible.

Unilateral middle cerebral artery occlusion (MCAO) in both humans and rodents induces contralateral neurological deficits [3] and a compensatory reliance on the less impaired side of the body ipsilateral to the injury [4]. Compensatory behaviors are developed in order to perform daily activities despite impairments [5]. Unilateral brain damage results in deficits of symmetry, therefore it is useful to rely on tests that have the ability to detect asymmetries [6]. Tests of asymmetry help factor out confounding variables such as overall decrease in activity after surgical induction of stroke [6]. Several problems may arise and be considered when selecting and performing functional evaluations, which include ethical considerations, availability of funding, choosing tests that are sensitive and the time available for task training, if necessary. Additionally, selecting the best times to test after surgery must be carefully taken into consideration. Animals, like humans, show spontaneous recovery, therefore it can be difficult to pinpoint and evaluate treatment effects.

The present aim of this paper is to review many of the current behavioral tests that assess functional outcome after stoke in the rodent model. The primary functions of the behavioral tests discussed are presented in Table 1 while factors associated with these tests are presented in Table 2. Most of the behavioral studies have been characterized in rodents that have undergone MCAO. There are many behavioral tests that are available for rodent stroke models, but no one test has stood out as superior to others in fully characterizing the various deficits that occur after stroke. Different tests are sensitive to measuring deficits associated with particular areas of damage. There are behavioral tests that assess both acute and chronic impairments in rodent models. Identifying behavioral and pharmaceutical interventions that will improve recovery from stroke damage is a fundamental component of stroke research. Translational approaches aimed at rehabilitation rely on the reliability and validity of animal paradigms and their ability to replicate the human model of stroke.

**Table 1:** Primary Functions of Assessments

Behavioral Test	Function
Composite Scores	Assesses a variety of motor, sensory, reflex and balance responses
Cylinder Test	Assesses spontaneous forelimb use
	
Grid Walking	Assesses sensorimotor function, motor coordination and placing deficits during locomotion
Ledged Tapered Beam	Assesses hindlimb functioning
	
Reaching Chamber/Pellet Retrieval	Assesses skilled forepaw use and motor functioning
Staircase Test	Assesses forelimb extension, grasping skills, side bias and independent use of forelimbs
Pasta Test	Assesses manual dexterity and fine motor skills
Ladder Rung Walking test	Assesses fore- and hindlimb stepping, Placing and coordination
Forelimb Flexion	Assesses forelimb function
	
Forelimb Placing	Assesses forelimb function and placing deficits
Corner Test	Assesses sensorimotor and postural asymmetries
Accelerated Rotarod	Assesses motor coordination and balance
	
Adhesive Removal	Assesses tactile responses and asymmetries
Morris Water Maze	Assesses spatial learning and memory
	
Radial Arm Maze	Assesses spatial learning and memory

**Table 2:** Factors Associated with Behavioral Testing

Behavioral Test	Approximate Training Time	Number of Trials Per Session	Approximate Time to Complete Test
Composite Scores	None	Dependent upon task	Dependent upon task
Cylinder Test	None	1 trial	2-5 minutes
Grid Walking	1 day	1-2 trial(s)	5 minutes
Ledged Tapered beam	2-3 days	5 trials	2-5 minutes
Reaching Chamber/	2-4 weeks	20-30 trials	5-10 minutes
Pellet Retrieval			
Staircase Test	2-4 Weeks	1-3 trials	5-15
Pasta Test	None (exposure to pasta 5 days before testing is best for optimal performance)	3-5 trials	10-20
Ladder Rung Walking Test	1 day	5 trials	10 minutes
Forelimb Flexion	None	1 trial	1 minute
Forelimb Placing	None	10 trials	5 minutes
Corner Test	None	10 trials	5-10 minutes
Accelerated Rotarod	2-4 days	3 trials	10 minutes
Adhesive Removal	None (exposure to pasta 5 days Before testing is best for optimal performance)	4-5 trials	5-10 minutes
Morris Water Maze	Dependent upon task	Dependent upon task	Dependent upon task
Radial Arm Maze	Dependent upon task	Dependent upon task	Dependent upon task

### Composite Scores

#### Bederson Scale and Neurological Scoring Scales

Following stroke, animals subsequently exhibit a variety of neurological deficits. The Bederson scale is a global neurological assessment that was developed to measure neurological impairments following stroke [7]. Tests include forelimb flexion, resistance to lateral push and circling behavior. A grading scale of 0-3 is used to assess behavioral deficits after stroke. This scoring scale is a simple way to reveal basic neurological deficits. Ischemic animals will have significantly more neurological deficits than non-ischemic animals, resulting in a higher score [3,8,9]. Many iteration scales have been developed and modified since the Bederson scale, all of them providing simple ways to detect impairments [9-11]. Although easy to perform, neurological ratings on this scale are limited because of their subjective nature. In addition, deficits on the Bederson scale resolve quickly in many common stroke models, rendering it less useful for the detection of long-term deficits after stroke.

#### Modified Neurological Severity Scores (mNSS)

One of the most common neurological scales used in animal studies of stroke is the modified neurological severity scores (mNSS). The mNSS rates neurological functioning on a scale of 14 or 18, depending on mice or rat, respectively. The mNSS includes a composite of motor (muscle status and abnormal movement), sensory (visual, tactile and proprioceptive), reflex and balance tests [3,12]. One point is given for the inability to perform each test while one point is deducted for the lack of a tested reflex, and an overall composite score is given to determine impairment. Neurological rating scores have the ability to assess multiple deficits and can be good for testing over periods of 30-60 days [13]. Despite the simplicity of administering the tasks, deficits may be specific to a certain modality or function and could be masked by the composite score. In addition, the reflexes tested in the mNSS are the pinna and startle reflexes, which are unlikely related to damage within the MCA territory.

### Motor Tests

#### Cylinder Test

**Figure 1:**
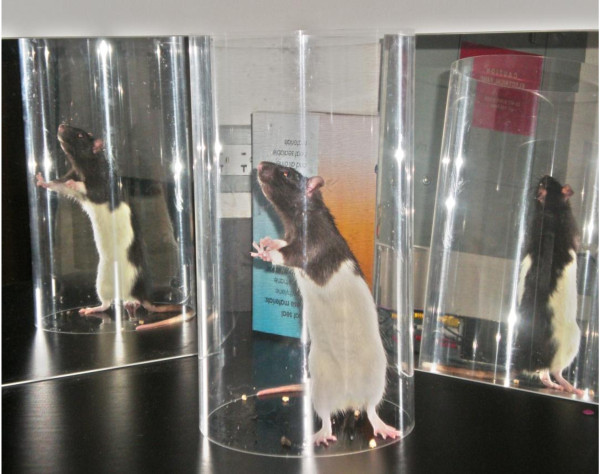
**Cylinder Test**. A rat's spontaneous forelimb use being assessed using the cylinder test.

Exploratory behavior in the rat provides a possibility to investigate the neural basis of spatial and motor behavior, which can be used as an assay of brain function [14]. The cylinder test (Figure 1) provides a way to evaluate a rodent's spontaneous forelimb use and has been used in a number of motor system injury models of stroke [15-19]. To evaluate forelimb deficits, the animal is placed in a transparent Plexiglas cylinder and observed. Rats will actively explore vertical surfaces by rearing up on their hindlimbs and exploring the surface with their forelimbs and vibrissae. When assessing behavior in the cylinder, the number of independent wall placements observed for the right forelimb, left forelimb and both forelimbs simultaneously are recorded. Animals with unilateral brain damage will display an asymmetry in forelimb use during vertical exploration [6]. The cylinder task has been found to be objective, easy to use and score, sensitive to chronic deficits that others fail to detect and have high inter-rater reliability [19]. In addition, no pre-training is required, although it is best to obtain baseline data to test for pre-operative bias because, on occasions, some animals display independent use of one limb [17]. It is best to use this assessment during the animal's dark cycle and under red lighting conditions because rodents are more apt to explore in a dark environment [19]. This test has also been found to have the ability to detect even mild neurological impairments [6].

#### Grid Walking

The grid walking task, often referred to as the foot fault task, is a relatively simple way to assess motor impairments of limb functioning (most commonly hindlimbs, but forelimbs have been evaluated as well) and placing deficits during locomotion in rodents. This task has been found to objectively demonstrate motor coordination deficits [2] and rehabilitation effects after stroke [5,12]. An animal is placed on an elevated, leveled grid with openings. Animals without brain damage will typically place their paws precisely on the wire frame to hold themselves while moving along the grid. Each time a paw slips through an open grid, a "foot fault" is recorded. The number of both contra- and ipsilateral faults for each limb is compared to the total number of steps taken and then scored using a foot fault index [15]. Intact animals will generally demonstrate few to no foot faults [2,20], and when faults occur, they do so symmetrically [20]. Ischemic animals typically make significantly more contralateral foot faults than intact animals [2,13,16,21]. The foot fault test has been shown to be a sensitive indicator for detecting impairments of sensorimotor function after ischemia in rodents [22] and requires very little pre-training [2,20]. However, the foot fault has not been shown to provide a good model for testing long-term deficits beyond 30 to 60 days [13]. This time-dependent test appears more appropriate for exploring early treatment effects due to spontaneous recovery [21].

#### Ledged Tapered Beam

Hindlimb testing in rats can be difficult because the hindlimbs in rodents are not generally used for complex movement [19]. The ledged tapered beam-walking test, however, has been used as a reliable measure of hindlimb functioning [19]. This test is a locomotor assessment that detects placement dysfunction of the hindlimbs after unilateral brain damage. In this task, animals must walk across an elevated balance beam that tapers at one end and has an under-hanging ledge. Foot faults made with hindlimbs are viewed as deficits in hindlimb function. After a stroke, an animal's foot faults will increase on the contralateral side as the ledge tapers and the difficulty of the task increases. For this task, pre-training is required until the animal successfully walks across the beam without turning around and without making any faults. It is also important to allow each animal a short break between trials so the animal does not habituate to the task [19]. Although hindlimb testing has been found to be rather difficult to assess in the animal model, the ledged tapered beam task reveals deficits that rats might normally make compensatory adjustments that they can conceal [19]. This assessment may be useful not only for determining the extent to which experimental therapies promote brain repair in but also may be helpful to show motor learning [4]. The ledged beam task might be more sensitive to detect hindlimb placing deficits while the grid walking test might be more sensitive to impairments of forelimb functioning [20].

#### Reaching Chamber/Pellet Retrieval Task

**Figure 2:**
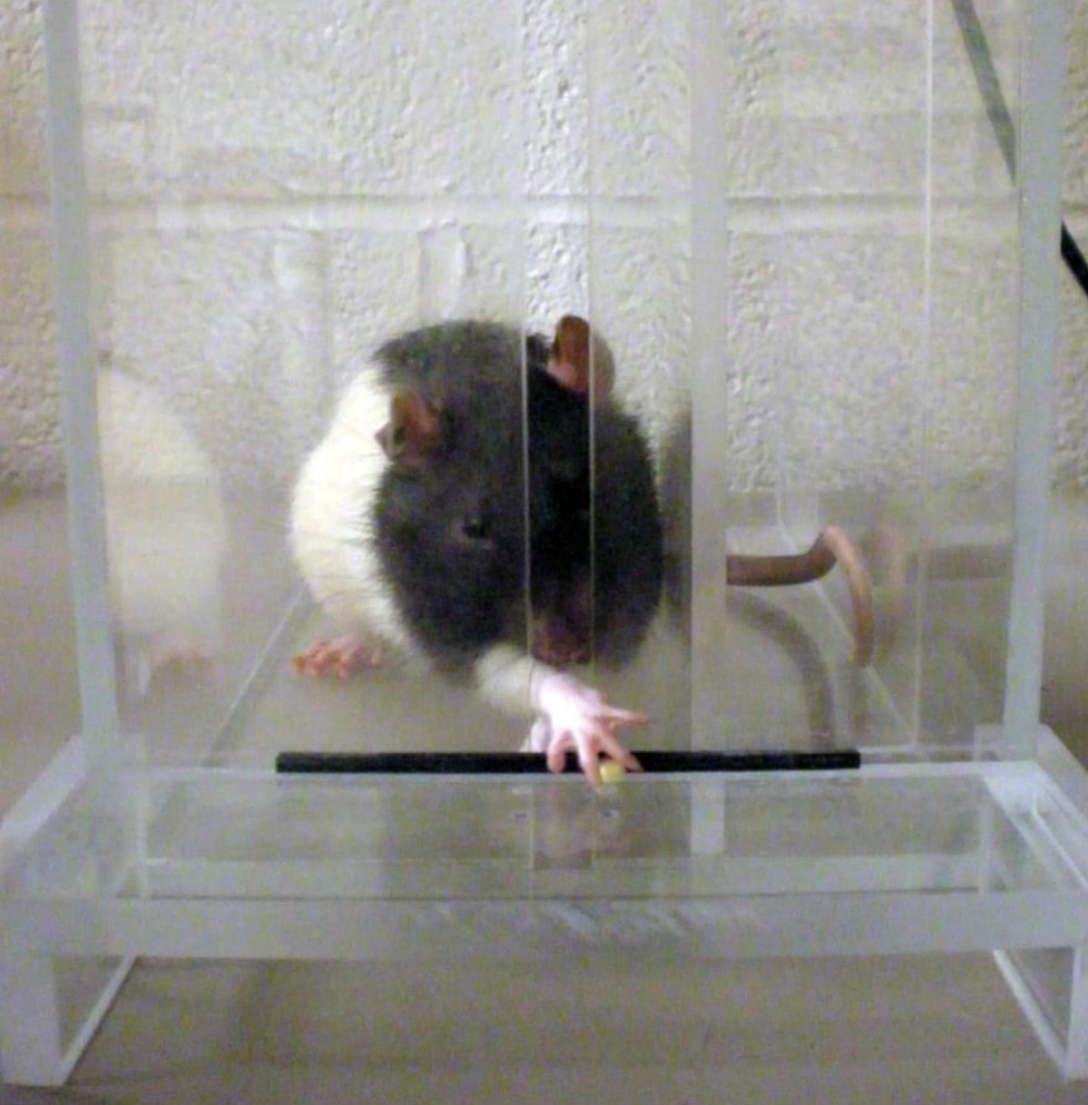
**Reaching Chamber/Pellet Retrieval**. A rat performing the single pellet retrieval task in a reaching chamber. The rat reaches for banana pellets located contralateral to the trained reaching limb.

It has recently been shown that rodent skilled paw movements are more similar to primate hand movements than once thought [23]. Reaching chambers have been useful for the investigation of skilled forepaw use and motor functioning deficits after unilateral brain damage in rodents [5,24,25]. The chamber consists of a Plexiglas apparatus with a tray located on the outside of the wall that holds food targets. The single pellet tray with two indentions can be used to examine an animal's success at retrieving an individual pellet. A pellet is placed in the indention contralateral to the reaching limb in order to evaluate unilateral brain impairments (Figure 2). A reaching tray can also be used to analyze the overall number of pellets retrieved in a given amount of time. A removable barrier, designed to inhibit pellet reaching from one side of the body, can be used for forced retrieval from either side. An animal must reach through a window, grasp and retract the pellet. Attempts, successes, failures and drops are recorded in order to determine impairments. In addition, time from reaching to eating and paw preference for reaching can also be measured. Damage to the motor cortex region from stroke results in impairments of skilled reaching [26,27].

The skilled reaching task has also been used to investigate the effects of intact forelimb usage on the recovery of the impaired forelimb after stroke [5]. Rats have been found to compensate for motor deficits displayed in skilled reaching by relying on the unimpaired limb [28]. By restraining the use of the unimpaired limb, a researcher can evaluate the effects of forced use of the impaired forelimb. In addition, altering the height of the single pellet tray has been shown to detect more subtle deficits such as postural adjustments [24]. Grasping and digit movements can also be observed after motor cortex damage from stroke [23]. Although paw dexterity, which is commonly impaired in human stroke patients, can be evaluated in this task, the rat paw is small and digit movements can be difficult to document.

Training for the skilled reaching task generally requires a long period of pre-training by first shaping the animals to acquire the skills of the task. In order for animals to be sufficiently motivated to participate in the task, animals are typically food deprived and kept at 85-90% of their normal body weight. Despite long periods of training, paw reaching is a valuable way to assess motor functioning because it is sensitive to movements that seem to be unimpaired in other voluntary behaviors [29], and rats' skilled forelimb movements are similar to those of humans [30]. Additionally, deficits can be detectable for up to three months [31].

#### Staircase Test

**Figure 3:**
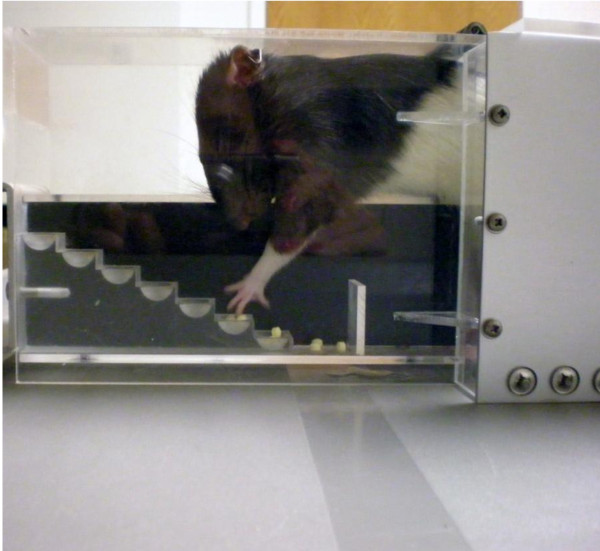
**Staircase Test**. A rat performing the staircase test. The rat extends its arms and reaches for banana pellets located in the wells.

The staircase test (Figure 3) was developed to assess the independent use of forelimbs of rats [32] and was later adopted to assess skilled reaching in mice [33]. This test allows for the bilateral measurement of an animal's forelimb extension, grasping skills and side bias by observing its behavior in a task requiring it to reach for food pellets. The apparatus is designed to encourage the animal to gain access to food by entering a narrow space, a natural rodent behavior [32]. After the animal climbs on a platform, it must reach to either side to retrieve food from a double set of staircases. Pellets are placed on each step on both sides. The animal cannot simply scoop the pellet; it must make a coordinated reach and grasp to retrieve it. A normal animal will typically collect the pellets rapidly [34]. Latency and the number of pellets from each side and location at increasing distances are then calculated to determine impairments. Animals will often attempt to reach and retrieve pellets but instead knock them to lower levels. To account for this, Adkins-Muir and Jones [35] have provided a useful improvement that allows the experimenter to know which pellets were grasped and retrieved and which were knocked to lower levels by staining the pellets different colors for specific staircase levels.

The staircase test allows for the assessment of forelimb reaching capacities, sensory capacities, dexterity and motor coordination [8]. It has been shown by some to be sensitive to detecting long-lasting deficits from ischemia [1,8]. A key advantage of this task is the ability to evaluate skilled use of the limbs independently following impairment. Although food deprivation and daily pre-training of a few weeks is generally required to teach the animals to retrieve the pellets [8,34], the test is easy to score [34]. One potential drawback, however, is that the test may not detect detailed behavior and it lacks sensitivity to forepaw preference compared to other tests [32]. Since rehabilitation efforts for unilateral brain damage are more sensitive when evaluating independent limb use of the contralateral limb as opposed to examining limb bias, the ability of the staircase test to evaluate independent limb functioning is a key feature [32]. This task has been found to be an effective way to examine functional motor capabilities and goal-directed paw use after ischemia [31,32].

#### Pasta Test

**Figure 4:**
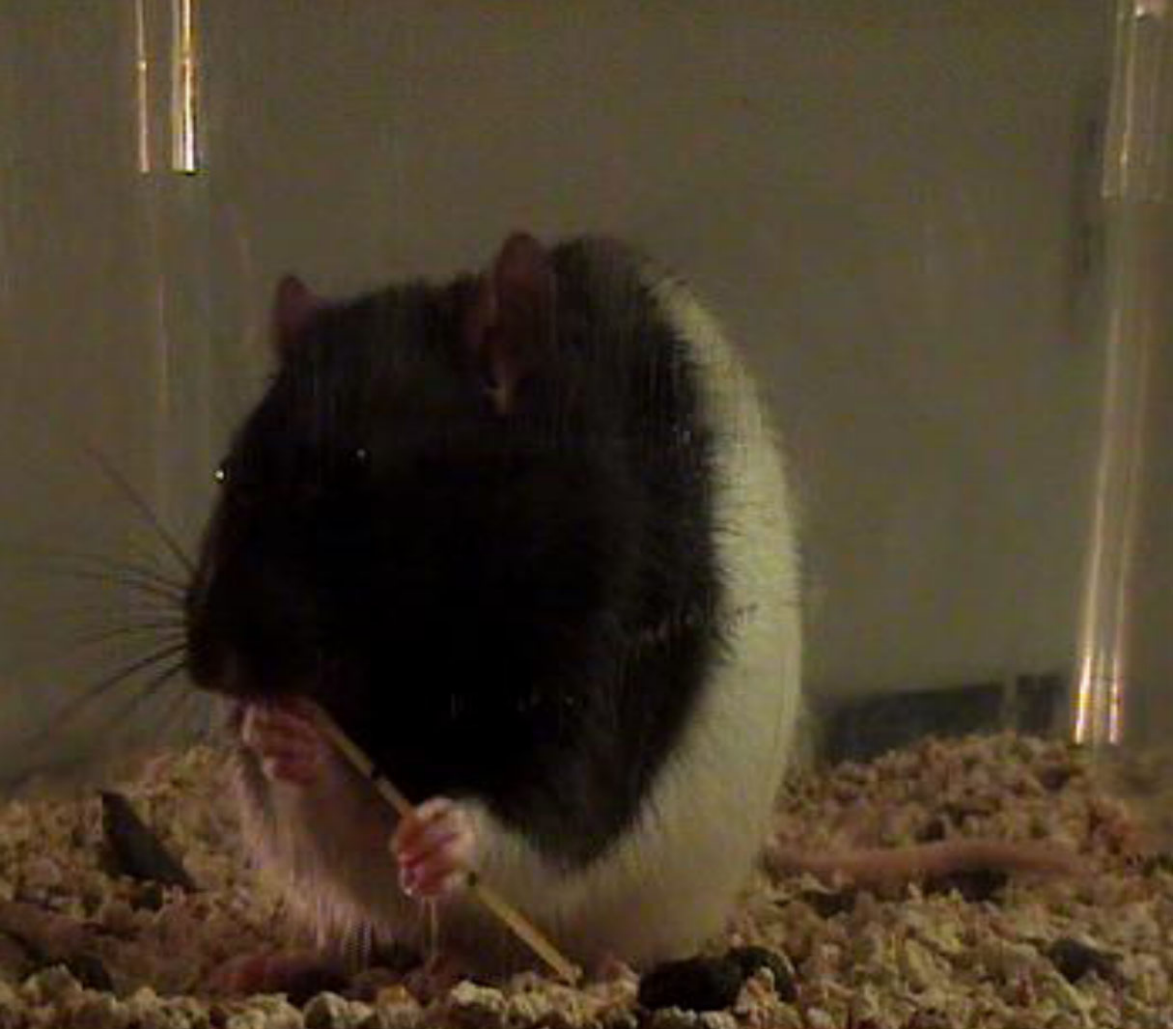
**Pasta Test**. A rat holds the pasta in an asymmetrical position, a typical behavior in a non-brain damaged rodent.

In addition to reaching, manual dexterity is often impaired after suffering damage to the central nervous system. Rats live in environments that require them to manipulate objects and use a wide array of motor skills to gain access to food [19]; thus dexterous manipulation of items are part of their repertoire. While there are only a few tests that have the ability to measure skillful forepaw use, the pasta test has been shown to reveal manipulative skilled impairments. When eating a piece of pasta, a rat's fine motor skills are often asymmetrical at first (Figure 4). It will use one paw to perform supportive functions and the other paw to move the piece of pasta. One paw, referred to as the "grasp paw", is used in a whole paw grasp and is typically positioned further away from the mouth. The other paw, referred to as the "guide paw", holds the pasta between one or two digits and the thumb, is positioned closer to the mouth and is used to guide the pasta into the mouth. Adjustments are made with both paws as the pasta is eaten. As the pasta becomes shorter, the paws move together into a symmetrical holding pattern. As symmetrical holding is acquired, one paw becomes directly placed on top of the other, and the digits become interposed. The number of adjustments for each paw is recorded as well as the number and type of atypical behaviors. After stroke, a rat will show differences in the number of adjustments made with each paw as well as an increase in atypical behaviors. This test is quantitatively measureable, relatively simple to administer and is sensitive to sensory, motor and lateralized impairments [36]. While other dexterous tasks can be labor intensive, the pasta test requires no shaping and little pre-training.

#### Ladder Rung Walking Test

The ladder rung test, initially developed for rats [37] and later adapted for mice [38], is another assessment used to assess skilled walking for both fore- and hindlimb placing. This test is designed to measure placing, stepping and inter-limb coordination [37]. The apparatus consists of a horizontal ladder which an animal spontaneously walks across. The spacing of the ladder rungs can be varied to prevent a compensatory reliance for impairments through learning the spacing and location of the rungs [37]. For analysis, video recordings of foot faults are generally scored on a rating scale according to quality of limb placement. Animals with motor system injury have been shown to display impairments in the ladder rung test after stroke [37-39]. This task requires minimal training and is useful for measuring chronic deficits and long-term treatments [37].

### Sensorimotor Tests

#### Forelimb Flexion

A very basic assessment used to detect neurological deficits is a test of forelimb flexion. This test requires an experimenter to suspend a rat by its tail. The posture of the forelimb is then observed and rated. Typically, an animal will extend its forelimbs toward the ground. A rat that has undergone MCAO will flex the contralateral forelimb and twist its body towards the contralateral side of damage [16,21]. Deficits will tend to decrease with time as recovery occurs. This measure has been found to be particularly sensitive to detecting deficits for several weeks following MCAO [16].

#### Forelimb Placing

A rat's vibrissae play a very important role in its sensory environment [19]. Forelimb placing can be assessed by stimulating a rat's vibrissae to trigger a response. To test forelimb function, an animal is held by its torso, forelimbs hanging free, while brushing its vibrissae on the corner edge of a table. Damage to the motor system will elicit paw placing impairments. Non-brain damaged animals will typically elicit a response of placing the forelimb ipsilateral to the side of vibrissae stimulation on the table [6,8,40]. This task can be scored as either calculating the percentage of placing responses for contralateral and ipsilateral responses [15,16] or on a scoring scale [8]. Animals with unilateral brain damage have been found to have difficulty eliciting a placing response on the contralateral side [6,17,40]. Intact rats will generally have a high success rate with this task [19]. Although the forelimb placing test has been found to detect even mild neurological impairments [6,16], a well-handled rat is required for accurate scoring. It is also important to have an experienced examiner in order to prevent abrupt movements resulting in an elicited response [4].

#### Corner Test

The corner test is a sensorimotor functional assessment that has been shown to be reliable for identifying and quantifying sensorimotor and postural asymmetries. It has also been shown to provide a simple way of detecting contralateral deficits and ipsilateral turning biases. This test was first described while investigating unilateral nigrostriatal damage in the rat [18] and was later used to investigate focal cerebral ischemia in the mouse [13]. The apparatus consists of two boards placed closely together at a 30 degree angle to form a narrow alley. An animal is then placed in between the boards facing the corner. As the animal approaches the corner, both sides of the vibrissae are simultaneously stimulated which leads the animal to rear and turn 180 degrees. Intact animals will usually turn around to the right or left randomly while animals with unilateral brain damage will preferentially turn around in the ipsilateral direction, leading with the non-impaired limb and displaying an asymmetry in corner turning [6,12,13]. Baseline data is recommended for the reduction of variability and identification of preferential side. In addition to identifying sensorimotor deficits, the corner test has been shown to be an objective assessment of long-term functional outcome (up to 90 days) after stroke in both the rat and the mouse [4]. The corner test might be more sensitive in detecting deficits than the other symmetry tests because it reflects multiple asymmetries, including forelimb, hindlimb, postural and turning bias [6]. Some feel that the corner test is a measurement that is equated with neglect in stroke patients.

#### Accelerated Rotarod

The rotarod test is used to assess motor coordination and balance alterations in rodents [1]. It was first introduced by Dunham and Miya [41] to study neurological functioning and was later improved to investigate motor deficits of naïve mice and the effects of drug administration [42]. Since then, many have used it to explore brain injury such as stroke [10]. The rotarod apparatus is a rod which rotates at an adjustable speed. The speed of the rod increases with time, and the amount of time the animal remains on the device is recorded. Animals with ischemia have been shown to have significantly shorter times of staying on the spinning rod [1,2]. For this test, a researcher only needs to devote a minimal amount of time to train each intact animal to perform this task before brain injury [2]. Human survivors of stroke often suffer from lack of coordination [2], and this behavioral assessment offers an approach to investigate coordination deficits in the animal model. However, since stroke patients are not challenged with similar devices such as rotarods to test their balance, the translational significance of the rotarod is unclear. Despite this, its sensitivity to detect motor impairments in other ischemic models has been well-established [1,42].

#### Adhesive Removal Test

First developed to investigate stimulus-directed movement asymmetries resulting from unilateral nigrostriatal damage [18], the adhesive removal test is now commonly used to investigate stroke related impairments of tactile extinction [3,8,21]. Two adhesive tapes of equal size are applied as bilateral tactile stimuli on the dorsal side of the paws, which rats will naturally remove from their body by grooming. Tactile responses are measured by recording the time of initial contact with both the ipsilateral and contralateral paws and how much time after each contact it takes the animal to remove the adhesive from each side. Time to contact and time to remove separate out sensory vs. motor deficits. Rats with unilateral brain damage typically develop a bias for both contacting and removing the adhesive from the unimpaired, ipsilateral limb first [4,18-21]. For this assessment, pre-training and baseline data are recommended to obtain optimal level of performance as well as identifying any pre-operative asymmetries [8,19]. It is also essential to maintain consistency in the animal's testing environment, usually in the home cage, because small changes may impact the functional outcome [19].

The adhesive removal test can also be used to measure an animal's sensory asymmetries. The magnitude of sensory asymmetry is measured by adjusting the ratio of the size of the adhesive tapes on each limb. The adhesive on the impaired limb is increased while the adhesive on the unimpaired limb is decreased. This process is repeated until it has been determined between which two levels a bias exists. The magnitude of asymmetry test is highly sensitive to even minor deficits and treatment effects [18]. Changes can be quantified over time to measure behavioral recovery [20] and reinstatement of symmetrical tactile sensation [17]. This test can reveal asymmetrical biases in stimulus-directed activity in brain damage of sensorimotor areas and long-lasting deficits after focal ischemia [8] and MCAO [21].

### Cognitive Tests

#### Morris Water Maze

The Morris water maze [43] is a commonly used cognitive and behavioral assessment tool that assesses spatial learning and various memory systems. The apparatus consists of a round pool filled with opaque water. Animals are required to swim to a submerged platform to escape the water. Initial heading angle, latency to reach the platform and path length can be analyzed to study swimming behaviors and determine memory impairments. It has been used to evaluate learning for both reference and working memory impairments after stroke [4,9,16]. Damage to particular regions of the forebrain can be detected by using various procedural variables of the water maze [4]. MCAO has been found by some to cause prolonged disturbance of spatial memories in rats using this task [9,16,21]. Additionally, the water maze has been shown to reveal long lasting impairments in rats [16] and detect memory impairments in mice [44]. Other researchers, however, have been unable to detect spatial deficits after stroke in both the rat [15,45] and mouse models [1]. Some have suggested that ischemic rats only show slight impairments in cognitive functioning of navigation because they use a non-spatial strategy to find the platform [46] while others state the discrepancy may be due to variability in protocols [1].

Modifications of the traditional use of the maze have been developed by moving the platform's location within a quadrant to assess spatial navigation memory and flexibility of learning [47]. An advantage to this method is the ability to detect subtle search strategy deficits by probing spatial memory early and continuously as the animals learn [4]. Using the platform moving procedure, stroke animals have been found to display deficits after learning [4]. The water maze is also known for producing highly quantifiable data. A primary advantage of using the water maze over other common behavioral mazes to test memory is that there are no olfactory trails for animals to use scent tracking to find the target. In addition, food deprivation is not required for motivational purposes.

#### Radial Arm Maze

The radial arm maze is another method to study learning behavior and memory impairments in an animal stroke model. This task is also sensitive to hippocampal damage. The apparatus consists of a central platform with eight or twelve radial arms projecting from the center. Food targets are placed at the end of the arms. Generally, animals must be at least partially food deprived to initiate motivation. Animals are provided with ample spatial cues for spatial orientation. When testing for reference memory, an animal is required to retain a memory for the procedures of the task. The stimulus-response association remains constant for all trials [48]. Working memory assessment, however, requires the animal to remember which arms (target arms change from trial to trial) previously contained the food targets for one trial. Ischemic animals have been found to make more errors than non-ischemic animals when searching for food [45,46]. Some studies have shown impairments in both reference and working memory [45,46] while others have found that ischemic rats showed more impairments of working memory than reference memory [48,49]. These findings suggest that spatial cognitive functioning is impaired after ischemia, but it is difficult to fully explain the increase of working memory errors over reference memory errors [46].

## Conclusions

Assessing functional outcome in preclinical studies of stroke has become increasingly recognized. The variety of deficits that accompany stroke requires a diversity of behavioral tests, from global to modality specific. Overall, tests should be sensitive to the location of injury, extent of damage, and beneficial treatment [17]. Although we did not cover all of the behavioral assessments in the rodent stroke model, we did cover many that are commonly performed. The chosen battery of functional assessments should be able to detect even mild impairments. Because there are critical periods that are best for detecting deficits, identifying assessment and rehabilitation times is essential. For the best chance of ensuring successful functional evaluation after stroke in a rodent model, it is important to obtain baseline data before experimental manipulations. In addition, for tasks that require pre-training, animals must be properly trained before surgery for dependable post-operative data. Furthermore, reducing the animal's stress and anxiety during testing is imperative to obtain reliable data [17]. This can be done by simply handling the animals on a regular basis and testing in the home cage. Reducing variability is another key element that must be taken into account. Reliability, consistency and accuracy are fundamental factors of sound research. Barth et al. [20] note that differences in results across studies of contralateral impairments may be due to different methodologies used (testing in home cage vs. novel environment). Consistency when using methods that have been shown to target impairments is imperative for successfully detecting deficits. Moreover, it is also important for the experimenter to be blind to treatment conditions to help eliminate bias.

With the need for a better understanding of mechanisms that promote recovery after stroke, researchers should continue to investigate correlates of behavioral outcome and histopathological analysis. Functional assessments can complement histological data when evaluating effects, outcome and treatment of stroke. Recovery mechanisms may vary depending on the region of brain damage [20]. Future research should continue to investigate behavioral assessments and functional outcome of stroke and their relation to particular areas of brain damage in order to better understand rehabilitation strategies in animal models that may be translated to human stroke patients. In addition to acute recovery from stroke, behavioral research should also focus on recovery of long-term deficits [1]. Long-term functional recovery is important for evaluating treatments focused on enhancing brain tissue plasticity after stroke [17]. Since rodents often show spontaneous recovery after focal ischemia, tests that show long-lasting deficits are relevant for the application of treatments in clinical situations [1,8]. We also encourage that future research should also assess similarities and differences between males and females, young and old, and different strains of rodents, as recovery in different groups is clinically relevant.

Animal models are designed to provide therapeutic relevance in preclinical trials. One of the most central aspects in deciding which tests to utilize is the researcher's knowledge and experience of balancing the advantages and disadvantages of each assessment relative to what is being investigated. Selection of appropriate functional assessments for individual studies is essential. It is important to use a combination of neurobehavioral tests that are sensitive to deficits in order to detect the array of impairments that occur after stroke.

## Competing interests

The authors declare that they have no competing interests.

## Authors' contributions

KS wrote the manuscript. MB contributed significantly to the writing and editing of the manuscript. SS conceived of the idea to write the review and supervised and edited the writing of this review. All authors read and approved the final manuscript.
